# REFINING BP MONITORING: IMPACT OF HYPERTENSION AND VARIABILITY ON COGNITIVE TEST PERFORMANCE IN OLDER ADULTS

**DOI:** 10.1093/geroni/igae098.4064

**Published:** 2024-12-31

**Authors:** Mitchell Roberts, Erica Sappington, Pruthvinath Jeripity Venkata, Roma Tremblay, Carla VandeWeerd

**Affiliations:** UF Health Precision Health Research Center- The Villages, The Villages, Florida, United States; UF Health Precision Health Research Center- The Villages, The Villages, Florida, United States; UF Health Precision Health Research Center- The Villages, The Villages, Florida, United States; UF Health Precision Health Research Center- The Villages, The Villages, Florida, United States; UF Health Precision Health Research Center- The Villages, The Villages, Florida, United States

## Abstract

Blood pressure (BP) management is crucial in mitigating cognitive decline, yet the relationship between hypertension and cognitive function is complex. To further explore this, data were collected on cognitive function (via MoCA), demographics, hypertension, and BP (measured 3 times concurrently) from older adults 50-89 between 5/27-7/19/24 at the UFHealth PHRC in The Villages (UFIRB202400472) as part of a broader study to test and refine Casana’s Heart Seat (THS) algorithm for passive in-home BP monitoring. MoCA scores as the dependent variable, were examined using multiple regression. Predictors included age, gender, hypertension (self-reported diagnosis, systolic >160mmHg), BP variability (standard deviation of 3 BP’s), and pulse pressure, adjusted for interaction effects and confounding variables. A cross-sectional analysis of 502 older adults (M=71.98, SD=6.77) revealed that 54.2% (n=272) self-reported hypertension, with 95% of this subgroup reporting antihypertensive medication use. The mean MoCA score for all participants was 25.7 (SD=2.4). Regression analyses identified age and hypertension as significant predictors of lower MoCA scores (β = -0.621, p =.004). Higher mean systolic pressure was linked to lower MoCA scores (β = -0.193, p <.001). Older age (80+) correlated with greater BP variability (β = 0.067, p <.001) however, variability in systolic (p=.569) and diastolic (p=.280) BP was not significantly associated with MoCA scores. These findings highlight the importance of managing hypertension to preserve cognitive health in older adults. Implications will be discussed in terms of policy and practice.

